# Electronic health record-based assessment of cardiovascular health: The stroke prevention in healthcare delivery environments (SPHERE) study

**DOI:** 10.1016/j.pmedr.2016.07.006

**Published:** 2016-07-13

**Authors:** Randi E. Foraker, Abigail B. Shoben, Marjorie M. Kelley, Albert M. Lai, Marcelo A. Lopetegui, Rebecca D. Jackson, Michael A. Langan, Philip R.O. Payne

**Affiliations:** a The Ohio State University College of Public Health, Columbus, OH 43210, United States; b The Ohio State University College of Medicine, Columbus, OH 43210, United States; c Clínica Alemana de Santiago, Universidad del Desarrollo, Santiago, Chile

**Keywords:** 95% CI, 95% confidence interval, ACC, American College of Cardiology, AHA, American Heart Association, CDS, clinical decision support, CVH, cardiovascular health, EHR, electronic health record, GEE, generalized estimation equation, OSUWMC, Ohio State University Wexner Medical Center, SD, standard deviation, SPHERE, stroke prevention in healthcare delivery environments, Prevention, Primary care, Medical informatics, Health outcomes, Disease management

## Abstract

< 3% of Americans have ideal cardiovascular health (CVH). The primary care encounter provides a setting in which to conduct patient-provider discussions of CVH. We implemented a CVH risk assessment, visualization, and decision-making tool that automatically populates with electronic health record (EHR) data during the encounter in order to encourage patient-centered CVH discussions among at-risk, yet under-treated, populations. We quantified five of the seven CVH behaviors and factors that were available in The Ohio State University Wexner Medical Center's EHR at baseline (May–July 2013) and compared values to those ascertained at one-year (May–July 2014) among intervention (*n* = 109) and control (*n* = 42) patients. The CVH of women in the intervention clinic improved relative to the metrics of body mass index (16% to 21% ideal) and diabetes (62% to 68% ideal), but not for smoking, total cholesterol, or blood pressure. Meanwhile, the CVH of women in the control clinic either held constant or worsened slightly as measured using those same metrics. Providers need easy-to-use tools at the point-of-care to help patients improve CVH. We demonstrated that the EHR could deliver such a tool using an existing American Heart Association framework, and we noted small improvements in CVH in our patient population. Future work is needed to assess how to best harness the potential of such tools in order to have the greatest impact on the CVH of a larger patient population.

## Highlights

• Use and adoption of health information technology advances quality in patient care.
• Healthcare systems need tools to enhance primary prevention at the point-of-care.
• Providers and patients have shared accountability for population health metrics.

## 1 Introduction

Poor cardiovascular health (CVH) is associated with an increased risk for cardiovascular (Foraker et al., 2016, Folsom et al., 2011, Rasmussen-Torvik et al., 2013, Kulshreshtha et al., 2013) and other chronic diseases (Lloyd-Jones, 2014). CVH is amenable to change via prevention efforts (Lloyd-Jones, 2014, Bambs and Reis, 2011, Foraker et al., 2012). Population-level approaches to reduce tobacco use and promote healthy dietary choices (Eyre et al., 2004) are needed to achieve the American Heart Association's (AHA's) goal of “improving the CVH of all Americans 20% by 2020” (Lloyd-Jones et al., 2010). Prevention discussions at the point-of-care and early CVH interventions in the primary care setting may enhance and reinforce population-level strategies to improve CVH for all Americans (Peiris et al., 2015).

The increasing need for high-quality, patient-centered documentation at the point-of-care places time constraints on primary care providers, thus limiting behavior modification counseling during a patient encounter (Haire-Joshu and Klein, 2011, Huang et al., 2004). Evidence suggests that patient-centeredness decreases as providers increase attention to an electronic health record (EHR) (Street et al., 2014). In fact, primary care providers spend almost as much time documenting the encounter as they do in direct patient care (Ammenwerth and Spötl, 2009). Further, CVH data are often located on various screens throughout the EHR, limiting the ability of care providers to synthesize and reason upon such data, particularly in time-constrained practice settings. Compounding this problem, many providers lack training in delivering prevention messages and supporting behavior change (Kushner, 2010, Gunther et al., 2012). In a similar manner, few current EHR platforms provide tailored healthcare communication functionality as an alternative to shared decision-making (Mantwill et al., 2015, O'Malley et al., 2015, Chrimes et al., 2014). As a result of these barriers, few providers discuss physical activity or other lifestyle changes with patients nor do patients receive adequate information support to enable or promote such healthy behaviors (Eakin et al., 2005).

Clinical decision support (CDS) within EHR systems helps providers with decision-making tasks about individual patients at the point-of-care (Berner, 2006), and modifies provider behavior by recommending specific actions or reminding providers of clinical care guidelines (Rothman et al., 2012), prompts smoking cessation counseling and referrals (Sharifi et al., 2014), facilitates goal-setting among pre-diabetics (Chrimes et al., 2014), lowers cholesterol (Zamora et al., 2013), and increases appropriate prescribing (Litvin et al., 2013). There is growing consensus for improved patient outcomes through implementation of EHR tools in cardiothoracic surgery (Razavi et al., 2014), specialty clinics, and primary care (Zamora et al., 2013).

In response to the preceding challenges and opportunities, we developed and evaluated a novel, easy-to-use, EHR-based CVH assessment tool for use in primary care that automatically populates with EHR data and renders an interactive visual display of a patient's CVH score (Foraker et al., 2014). We hypothesized that the CVH of patients with access to our CVH tool would improve over a one-year period, while the CVH of patients without access to the tool would stay the same or worsen.

## 2 Material and methods

As we have described previously (Foraker et al., 2014), the stroke prevention in healthcare delivery environments (SPHERE) tool was developed and implemented in the outpatient EHR of a general internal medicine clinic at The Ohio State University Wexner Medical Center (OSUWMC) (Foraker et al., 2014). Briefly, the SPHERE tool (Supplementary Figure) was designed to increase patient-provider communication around prevention. The SPHERE tool launches within the EHR during a patient encounter, and is viewable by both patient and provider. The provider uses the interactive features of the tool (slider bars and buttons) to show how changes in each CVH component can impact their overall CVH. Providers in the intervention clinic were not incentivized to use the tool; details on its use are presented in a separate publication (Foraker et al., 2015).

Our eligible patient population included women who were 65 years of age or older at the time of the baseline encounter. Control clinic patients were seen in a different outpatient clinic in the OSUWMC system, and received usual care with regard to prevention discussions. The study, with a waiver of informed consent, was approved by The Ohio State University's Institutional Review Board (approval number 2013H0083).

Baseline demographic and CVH data for the eligible patient population at both clinics were obtained from the EHR for the time period of May 1, 2013 through July 31, 2013. The SPHERE tool was accessible to providers in the intervention clinic beginning October 6, 2013. Follow-up CVH data were queried one-year later for encounters occurring between May 1, 2014 and July 31, 2014. We conducted a group-level analysis on the subset of women seen in the intervention clinic (n = 109) and women seen in the control clinic (n = 42) who had an encounter during both time periods. We also report on the CVH of all eligible patients seen during baseline and/or follow-up periods. Baseline data indicated that 160 eligible patients were seen in the intervention clinic, and 62 eligible patients were seen in the control clinic. Follow-up data collection yielded 168 eligible intervention patients and 96 eligible control patients.

Baseline and follow-up data included the demographic variables of age and race (white, black, other). We used the most recent data (collected within the past 12 months) to characterize smoking status, body mass index (BMI), total cholesterol, blood pressure, and fasting glucose/hemoglobin A1c. Two other components of CVH, physical activity and diet, were infrequently entered as unstructured data in clinic notes, and were not computationally actionable as free text. Therefore, we excluded these two variables from the current study report.

We assigned patients to ideal, intermediate, and poor categories of the AHA's CVH metric as shown in Table 1, based on values associated with each of the biometric components, with the exception of fasting glucose/hemoglobin A1c (American Diabetes Association, 2012). For fasting glucose, we categorized participants into ideal (not taking glucose-lowering medication) and intermediate (taking glucose-lowering medication) CVH categories due to high levels of missing data for both fasting glucose and hemoglobin A1c laboratory values in our EHR.

### 2.1 Statistical methods

Demographic factors in both control and intervention clinic were characterized as mean (SD) for continuous variables and as proportions for categorical variables. For each of the five CVH categories, the proportion of women in each clinic in each category (e.g., ideal, intermediate, or poor health or missing) was reported both at baseline and post-intervention. Overall CVH score, calculated as 2 points for idea, 1 point for intermediate, and 0 for poor on each of the five available factors was calculated for each participant and the change in average CVH score in aggregate (pre-post) for each clinic was estimated using a GEE approach to account for correlations on women measured during both periods. The main analysis comprised the subset of women who were observed during both baseline and follow-up periods. A secondary analysis investigated changes among all eligible women seen during baseline and/or follow-up.

Data analysis was conducted using STATA (StataCorp). Statistical significance was set at 0.05.

## 3 Results

At baseline, the average age of the subset of patients seen in the intervention clinic was 75 years, while the average age was 72 in the control clinic (Table 2). Differences were seen at baseline by race between the intervention (35% black) and control (21% black) clinics. The demographic data in the intervention clinic did not change appreciably between the baseline and follow-up periods in either clinic. The baseline data of all eligible women seen during the baseline and/or follow-up periods in the intervention clinic had a similar distribution of demographic factors compared to the subset of patients at baseline. Similarly, the subset of patients in the control clinic had nearly equivalent demographic characteristics compared to all eligible women seen during the baseline and/or follow-up periods in the control clinic (Table 2).

Among women in the intervention clinic who were seen during both the baseline and follow-up periods, we observed CVH improve on the metrics of BMI (14.7 to 19.3% ideal) and diabetes (56.9 to 62.4% ideal) from baseline to follow-up. Meanwhile, the CVH of women in the control clinic either held constant (diabetes; 83.3% ideal) or worsened slightly (BMI; 23.8 to 19.0% ideal) from baseline to follow-up.

**Fig. 1:**
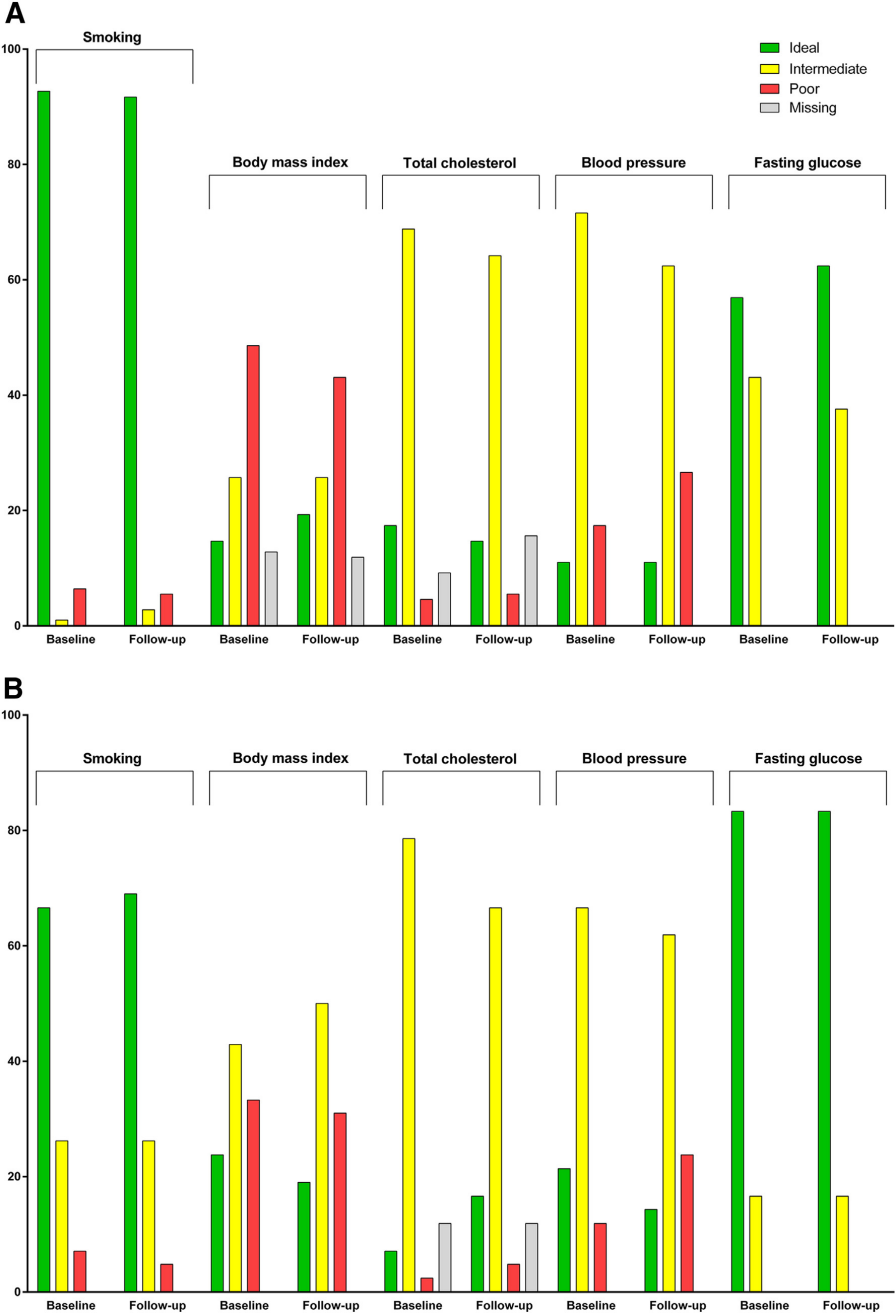

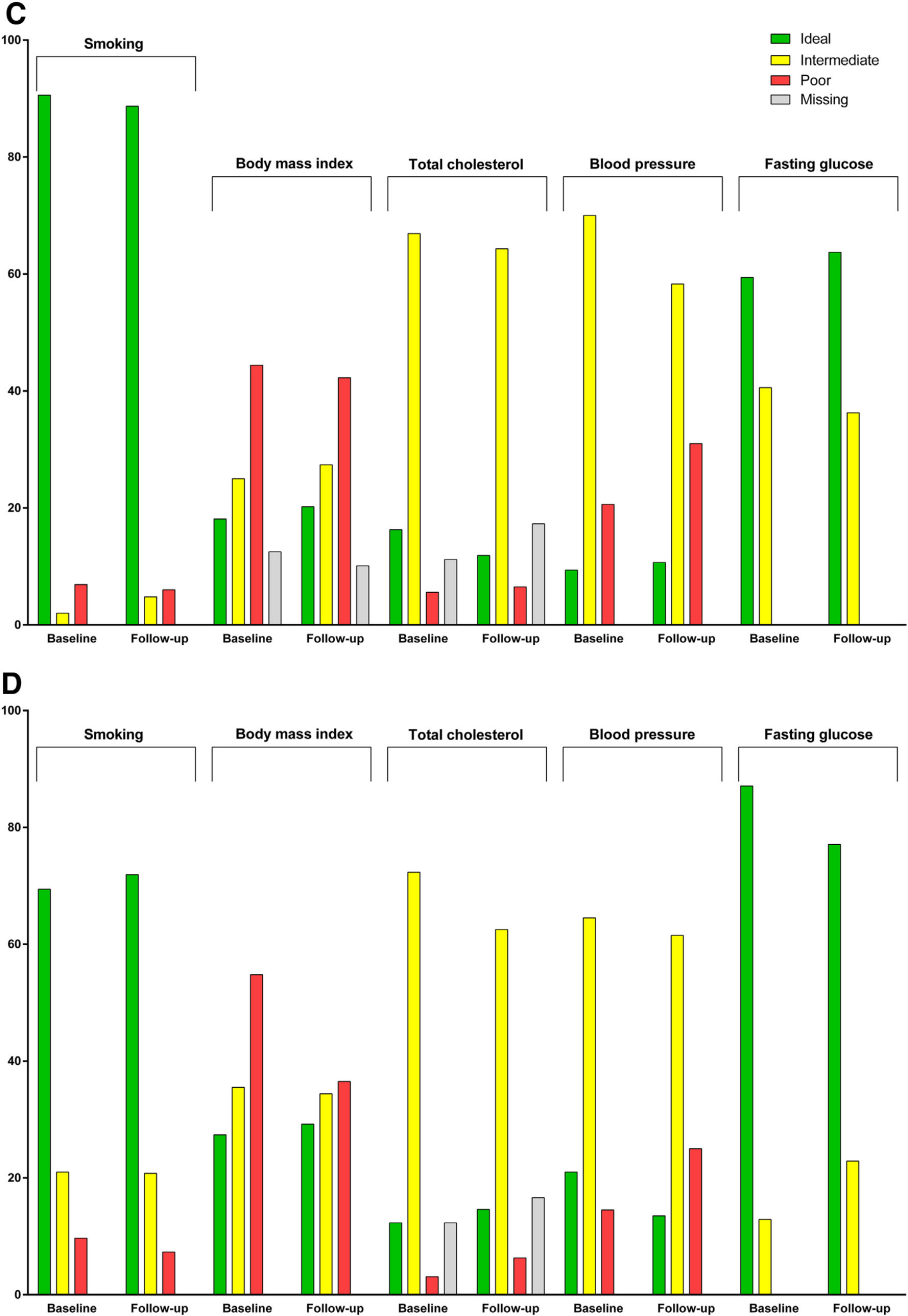
Changes in CVH from baseline (May–July 2013) to follow-up (May–July 2014) in the OSUWMC (A) intervention and (B) control clinics: patients seen in both baseline and follow-up periods. Changes in CVH from baseline to follow-up in the (C) intervention and (D) control clinics: patients seen in baseline and/or follow-up periods.

At baseline, a greater proportion of all eligible patients in the intervention clinic (Fig. 1C) were in ideal CVH compared to those in the control clinic (Fig. 1D) for current smoking and total cholesterol. However, eligible patients in the intervention clinic were more likely to be in poor CVH for BMI and blood pressure, and to be treated for diabetes, compared to the control clinic. Among all eligible patients in the intervention clinic, improvements were seen from baseline to follow-up for BMI (18.1 to 20.2% ideal) and diabetes status (59.4 to 63.7% ideal).

Average overall CVH score increased by 0.024 (95% CI: − 0.24 to 0.29) in the intervention clinic (p = 0.86), indicating that improvements in BMI and diabetes were somewhat offset by losses on other factors. Conversely, CVH components either held constant or worsened among eligible patients seen in the control clinic. In the control clinic, the estimated change in total CVH score was 0.018 (95% CI: − 0.40 to 0.44).

## 4 Discussion

In this relatively small sample of patients, we noted improvements in CVH, which has important clinical implications for the prevention of chronic disease (Lloyd-Jones, 2014). In particular, the SPHERE tool's focus on improving the CVH of patients in primary care is consistent with prevention services covered by the Affordable Care Act. These services include blood pressure, cholesterol, and diabetes screening; diet counseling; obesity screening and counseling; and tobacco screening and cessation support. As a result, the use of a CDS tool with a focus on prevention, like SPHERE, is responsive to the Stage 2 Meaningful Use criteria for EHRs (HealthIT.gov, 2011a, HealthIT.gov, 2011b, HealthIT.gov, 2013).

The SPHERE tool, by design, brings lifestyle factors into the workflow of patient care. As a result, the EHR becomes less of a passive data capture system as is typically the case, and instead, serves as the basis for timely and efficient shared decision-making. Shared decision-making allows for patients' values, needs, and preferences to guide evidence-based care (Barry and Edgman-Levitan, 2012), and our EHR systems should help improve shared decision-making around prevention of chronic disease (Kite et al., 2014). A Cochrane review of interventions for improving the adoption of shared decision making concluded that interventions targeting the provider and patient were more effective than those targeting the provider or patient alone (Legare et al., 2010).

To that end, providers need easy-to-use tools at the point-of-care to help patients improve CVH, as < 3% of Americans described in population-based studies are in ideal CVH according to AHA's metric (Foraker et al., 2016, Folsom et al., 2011, Kulshreshtha et al., 2013, Huffman et al., 2012, Bambs et al., 2011). We demonstrated that the EHR could deliver such a tool using an existing AHA framework designed to improve CVH (Lloyd-Jones et al., 2010). The success of the tool is due to ease of use, seamless integration with clinical workflow, functionality within the EHR window, non-intrusive design, and the automatic rendering of a CVH calculation at the point-of-care to improve patient-provider communication around CVH (Foraker et al., 2015).

A unique strength of the study was the use of an intervention clinic that was located in an underserved area of our community with a more racially diverse patient population as compared to the control clinic (Foraker et al., 2014). Our results add to a paucity of literature that demonstrates improvements in CVH using EHR-based tools (Mann and Lin, 2012). Unlike existing research, our study was done in a primary care setting and targeted all patients meeting age and clinic criteria, regardless of presence of comorbid conditions. Also distinct from other point-of-care interventions, the SPHERE tool is designed to be platform-independent and scalable to other patient populations and healthcare settings, not to mention EHR platforms (Foraker et al., 2014, Kite et al., 2014).

Limitations of this initial study stem primarily from the limited number of observations for older women seen in two outpatient clinics at baseline and again at one-year follow-up. Our data do not describe how many women initiated evidence-based treatment or who had laboratory assessments ordered as a result of their primary care encounter. In addition, diet and physical activity data were not recorded in the EHR. While we collected these data via the patient-facing EHR portal (56% response rate), these data were only collected at follow-up, not at baseline (Foraker et al., 2014). In addition, we were unable to detect changes in smoking behavior in the intervention clinic due to an already high prevalence of ideal smoking behavior in this clinic and the small sample size included in both baseline and follow-up periods.

Our study overlapped with another clinic-level intervention which targeted obese patients in the intervention clinic. The goal of that study was to encourage eligible patients to talk to their provider about their weight via an educational video. The medical assistant was to play the video for the patient while they waited for their provider in the exam room prior to their appointment. Of the patients eligible for that study, queried from the EHR from May 2012 through April 2013, 36 were females 65 years of age and older. Since the results of that study are not available, we cannot comment on the proportion of those 36 women who were shown the video while they waited for their provider, and whether they were the same women who were also eligible for the SPHERE study during a similar time period.

## 5 Conclusions

This is the first study to develop and implement an EHR-based CVH visualization tool. Our study demonstrates that it is feasible to implement patient-centered EHR-based tools at the point-of-care in the primary care setting. Our work answers the call for next-generation EHR design to facilitate patient-provider communication (Street et al., 2014). The SPHERE study is a unique multidisciplinary collaboration aimed at addressing the feasibility of using a system embedded in the EHR to improve patient-centered care. Future work is needed to assess how to best harness the potential of such tools in order to have the greatest impact on the CVH of a patient population.

The following is the supplementary figure related to this article.

Supplementary Figure SPHERE tool. Supplementary Figure

## Declaration of competing interests

The authors declare that there are no conflicts of interest.

## Funding sources

This study was funded by Pfizer Inc. (46214).

## Figures and Tables

**Table 1:** Measures of CVH according to the American Heart Association (Lloyd-Jones et al., 2010), and cut points used for hemoglobin A1c (American Diabetes Association, 2012).

	Poor health	Intermediate health	Ideal health
Smoking status	Yes	Former ≤ 12 months	Never or quit > 12 months
Body mass index	≥ 30 kg/m^2^	25–29.9 kg/m^2^	< 25 kg/m^2^
Total cholesterol	≥ 240 mg/dL	200–239 mg/dL or treated to goal	< 200 mg/dL
Blood pressure	Systolic ≥ 140 mm Hg or Diastolic ≥ 90 mm Hg	Systolic 120–139 mm Hg or Diastolic 80–89 mm Hg or treated to goal	Systolic < 120 mm Hg Diastolic < 80 mm Hg
Fasting glucose	≥ 126 mg/dL	100–125 mg/dL or treated to goal	< 100 mg/dL
Hemoglobin A1c	≥ 6.5%	5.7–6.4% or treated to goal	< 5.7%

**Table 2:** Demographic characteristics of all eligible patients seen at baseline (May–July, 2013) and follow-up (May–July 2014): OSUWMC.

	Intervention clinic			Control clinic		
Baseline (all eligible patients)	Follow-up (all eligible patients)	Baseline (patient subset*)	Baseline (all eligible patients)	Follow-up (all eligible patients)	Baseline (patient subset^a^)
N	160	168	109	62	96	42
Age (SD)	74.2 (6.7)	74.5 (7.0)	75.0 (6.8)	72.8 (7.5)	71.6 (6.7)	72.4 (7.4)
Race						
White	93 (59%)	96 (57%)	64 (59%)	45 (73%)	76 (79%)	30 (71%)
Black	56 (35%)	62 (37%)	38 (35%)	12 (19%)	14 (15%)	9 (21%)
Other	9 (6%)	9 (5%)	6 (6%)	5 (8%)	6 (6%)	3 (7%)

a Subset comprises patients seen in both baseline and follow-up periods.
