# Supplementary figures and images for: Electronic health record-based assessment of cardiovascular health: The stroke prevention in healthcare delivery environments (SPHERE) study
Source: Prev Med Rep. 2016 Jul 13;4:303–8. doi: 10.1016/j.pmedr.2016.07.006 (PMC4959947; doi:10.1016/j.pmedr.2016.07.006)

**Supplementary Figure. SPHERE tool.**


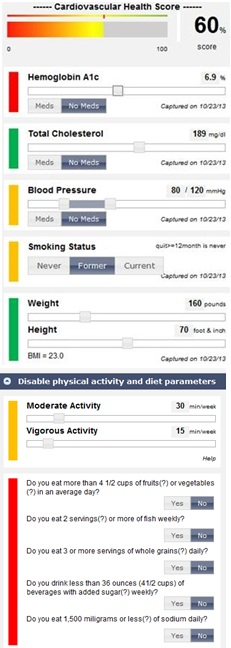

Supplement: Supplementary Figure — SPHERE tool. [file mmc1.docx]
